# Supplementary material for: Resistin Induces Migration and Invasion in PC3 Prostate Cancer Cells: Role of Extracellular Vesicles
Source: Life (Basel). 2023 Dec 10;13(12):2321. doi: 10.3390/life13122321 (PMC10744490; doi:10.3390/life13122321)
Supplement: Supplementary file 1 [file life-13-02321-s001.zip › life-2424944-supplementary.pdf]

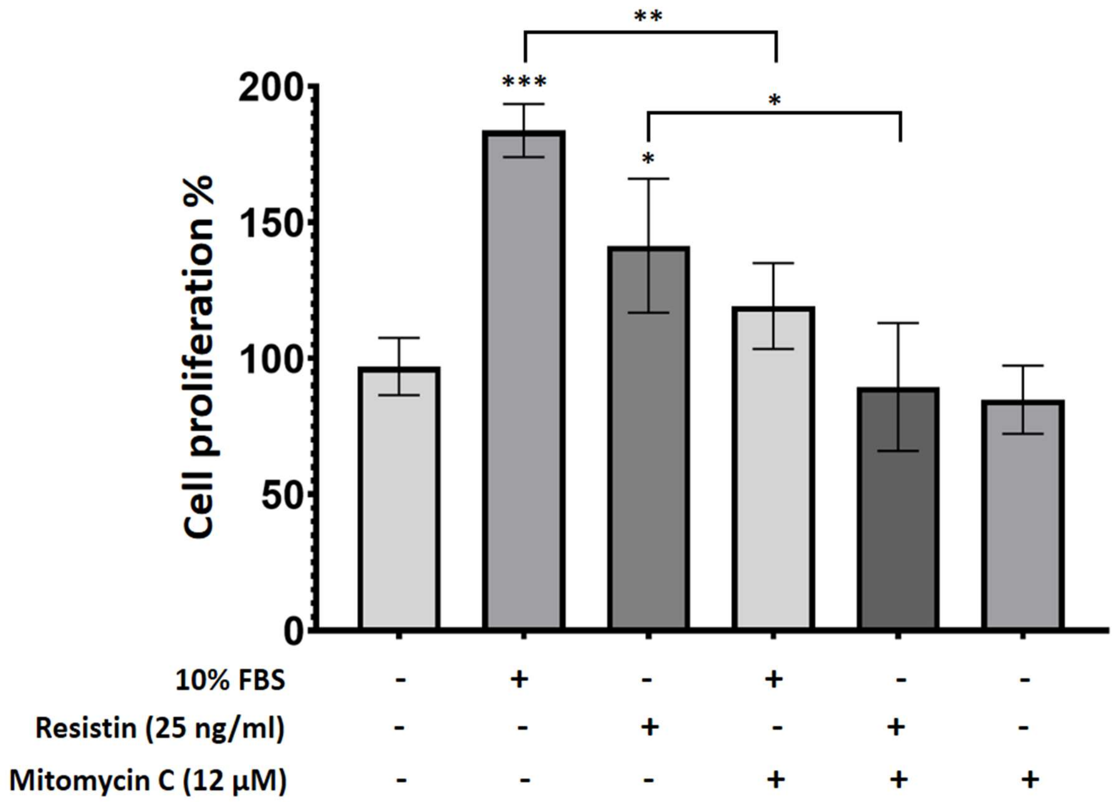

Supplementary Figure S1. Effect of resistin and mitomycin C on the proliferation of PC3 cells. Cell proliferation was evaluated at 48 h using the MTT assay. * $p < 0.05$, ** $p < 0.01$, and *** $p < 0.001$.

MTT Assay

Cell viability was assessed by an MTT reduction assay under different conditions for 24 h. First, cells were seeded onto 96-well plates at a 150,000 cells/well density and allowed to grow at 90% of confluence. Next, the culture medium was replaced with an Opti-MEM medium. After 2 h under this condition, cells were treated with several stimuli. Later, 30 µL of an MTT 2.1 mg/mL stock solution was added to the culture medium to obtain a final concentration of 0.5 mg/mL. Formazan crystals formed after 4 h of incubation and were further dissolved by adding buffer lysis (20% sodium dodecyl sulfate, 50% N,N-dimethylformamide, pH 4.0). Finally, optical density was measured at 570 nm using a microplate reader.

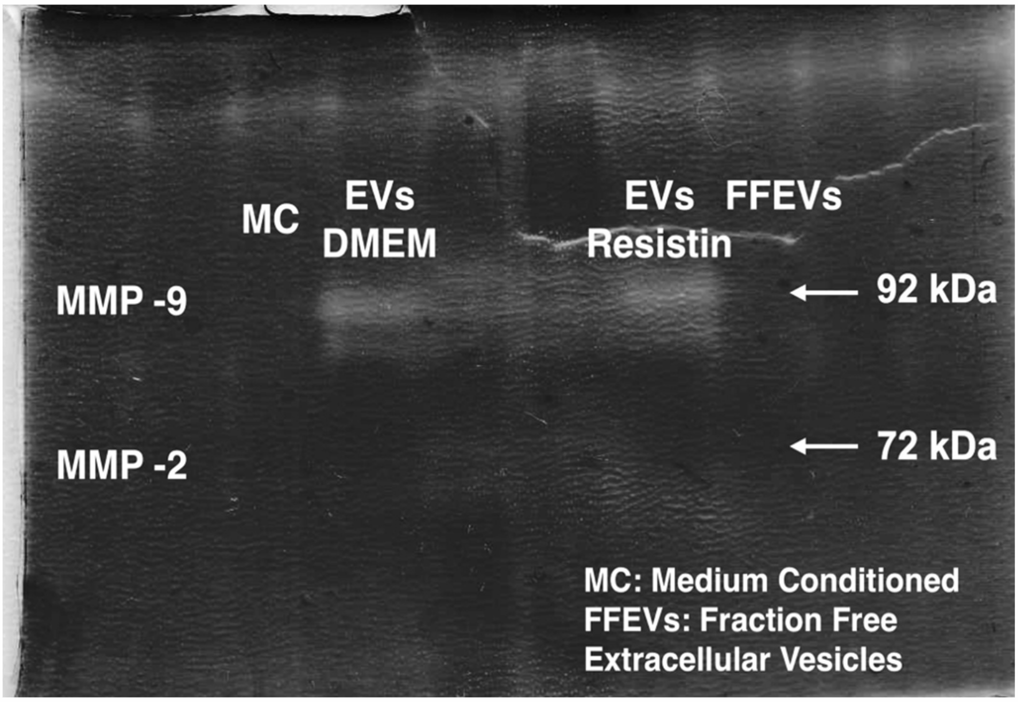

Supplementary Figure S2. Analysis of EVs fractions by zymography. Protein levels in EVs were quantified by micro-Bradford protein assay. In each condition, 25 µg of protein were analyzed.

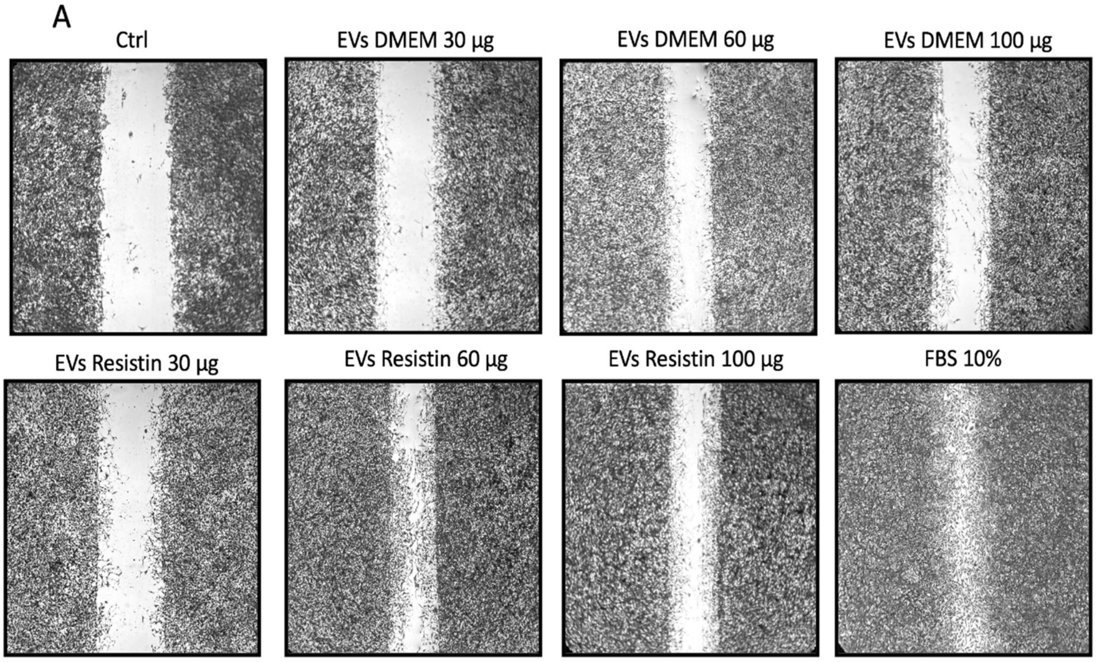

Supplementary Figure S3. The effect of resistin EVs on migration is dependent on protein levels. Cell migration was evaluated through scratch-wound assay. Cells were treated with different protein levels from EVs fraction (30, 60 and 100 µg/ml) and migration of cells was analyzed after 48 h.
